# Removal of an incarcerated intrauterine device reaching the serosal surface of the uterus by hysteroscopy alone: a case report

**DOI:** 10.3389/fmed.2024.1486745

**Published:** 2025-01-07

**Authors:** Min You, Qin-Fang Chen, Hai-Qian Lu

**Affiliations:** ^1^Department of Gynecology, Longhua Hospital, Shanghai University of Traditional Chinese Medicine, Shanghai, China; ^2^Department of Gynecology and Obstetrics, International Peace Maternity and Child Health Hospital Shanghai Jiao Tong University School of Medicine, Shanghai Key Laboratory of Embryo Original Diseases, Shanghai Municipal Key Clinical Specialty, Shanghai, China

**Keywords:** complications, intrauterine devices, incarceration, hysteroscopy, postmenopausal women

## Abstract

**Background:**

An intrauterine device (IUD) is a widely used long-term contraceptive device for family planning. However, the IUD can lead to various complications. Severe complications and remedial measures caused by IUDs have been reported in the literature; however, detailed surgical approaches for safely removing the IUD within the minimum surgical range have rarely been described especially in postmenopausal women. Therefore, this article aims to share our surgical experience in removing an IUD that had reached the serosal surface of the uterus using hysteroscopy alone after menopause to provide new clinical ideas.

**Case introduction:**

We report the case of a 63-year-old Chinese patient with a 12-year history of menopause. She had an IUD placed after an abortion more than 30 years ago. She came to the hospital because of occasional a small amount of unprovoked vaginal bleeding, the preoperative examination suggested an embedded IUD that appeared to have reached the serosal surface of the uterus. The IUD was not visible during hysteroscopic surgery because of uterine adhesions. Microscissors were employed to cut along the white adhesion band, revealing a faintly visible metal wire. We successfully removed the IUD using hysteroscopy only. The patient has recovered well after surgery and has been in good health for more than 5 months, with no complaints of abdominal pain or vaginal bleeding.

**Conclusion:**

This case suggests that hysteroscopic exploration can be performed in patients whose preoperative examination indicates that the IUD has reached or protrudes from the serosal surface of the uterus. If necessary, laparoscopic or open surgery can be performed. For patients whose IUD is not visible in the uterine cavity, preoperative imaging can help assess the thickness of the uterine myometrium and the distance to the serosal surface. Intraoperatively, scissors can cut through tissue or adhesions, and instruments can measure the separation distance or visualize the device within the adhesions. In addition, it is crucial to know the patient’s expectations, assess the pros and cons, and discontinue the procedure if necessary.

## Introduction

An intrauterine device (IUD) is a contraceptive device placed in the uterine cavity. It is widely used worldwide as a long-acting contraceptive due to its safety, cost-effectiveness, high efficiency, and reversibility. According to the Fourth International Conference on IUDs, more than 100 million people use IUDs globally, with more than 80 million in China, accounting for approximately 40% of contraceptive use among women of reproductive age (1).

However, in addition to contraceptive failure, IUDs can lead to complications, including ectopic pregnancy, detachment, and uterine incarceration (2, 3). Incarceration is the most common complication, with perforation due to incarceration and the consequences of damage to adjacent organs being potentially more severe; its incidence has been reported to be between 0.2 and 3.6 per 1,000 (3–5). Therefore, for women who choose IUD contraception, how to avoid the occurrence of complications, especially for postmenopausal women, and how to safely remove the IUD while preventing complications such as IUD incarceration due to organ atrophy have become challenging problems in clinical practice. Serious complications related to IUDs and their remedial measures have been reported in the literature (6), but for IUD incarceration, especially in postmenopausal women, employing the least damaging surgical technique to remove the IUD completely and safely is rarely described in detail. Therefore, in this study, we share our surgical experience in removing an incarcerated IUD that reached the serosal surface of the uterus by using hysteroscopy alone to provide new clinical diagnosis and treatment ideas.

## Case presentation

**Figure 1:**
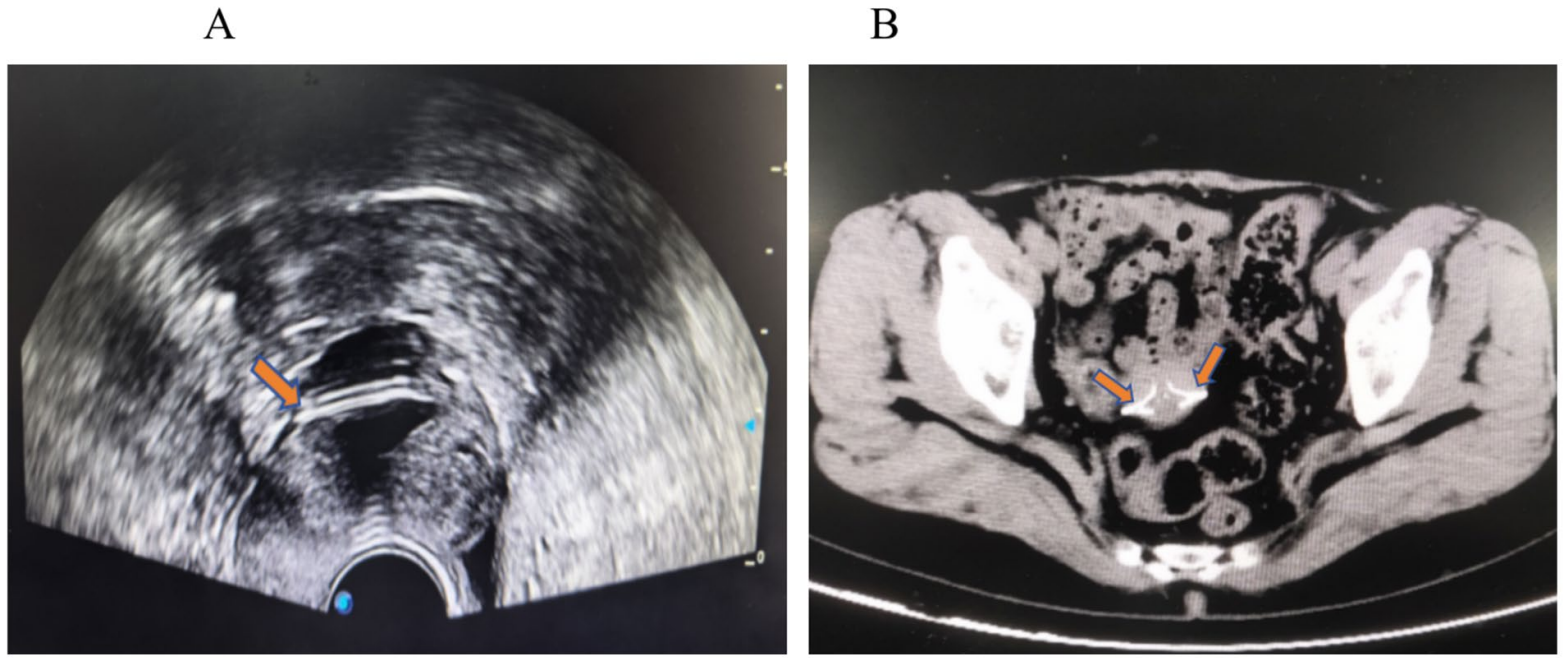
**(A)** Gynecological ultrasound: the right side of the IUD appears to reach the extraserosal aspect of the uterus. **(B)** Pelvic CT: the IUD is incarcerated, with edges protruding from the serosal surface of the uterus.

A 63-year-old Chinese woman presented with a 12-year history of menopause, 1-0-1-1, spontaneous delivery. She had an IUD placed after an abortion more than 30 years ago. The patient reported having regular menses lasting 3/30 days and no physical examination after menopause. The past surgical history included vaginal delivery and IUD placement after abortion, with no other past medical history. In 2022 and March 2023, she experienced a small amount of unprovoked vaginal bleeding for several days, neither of which was seen by a physician. Over the last 6 months, she had occasional small amounts of reddish vaginal discharge. In January 2024, an ultrasound performed at an outside hospital revealed an incarcerated V-type copper IUD and possible uterine fluid. In March 2024, she came to our hospital. She underwent an ultrasound, which showed a posterior uterus measuring 32 mm in length, 40 mm in width, 28 mm in thickness, with a single layer of endometrium measuring 2.2 mm in thickness, a 6.5 mm in uterine cavity separation, and 22 mm in long diameter of the cervix. The IUD was located in the myometrium of the right anterior wall and appeared to reach the extraserosa on the right side (Figure 1A). No free pelvic effusion was noted. A pelvic computed tomography (CT) scan revealed an incarcerated IUD with margins protruding from the serosal surface (Figure 1B). Gynecological examination revealed the following: cervix atrophy, light, pinpoint appearance of the external orifice; corpus uteri: posterior position, atrophy; and adnexa: negative.

**Figure 2:**
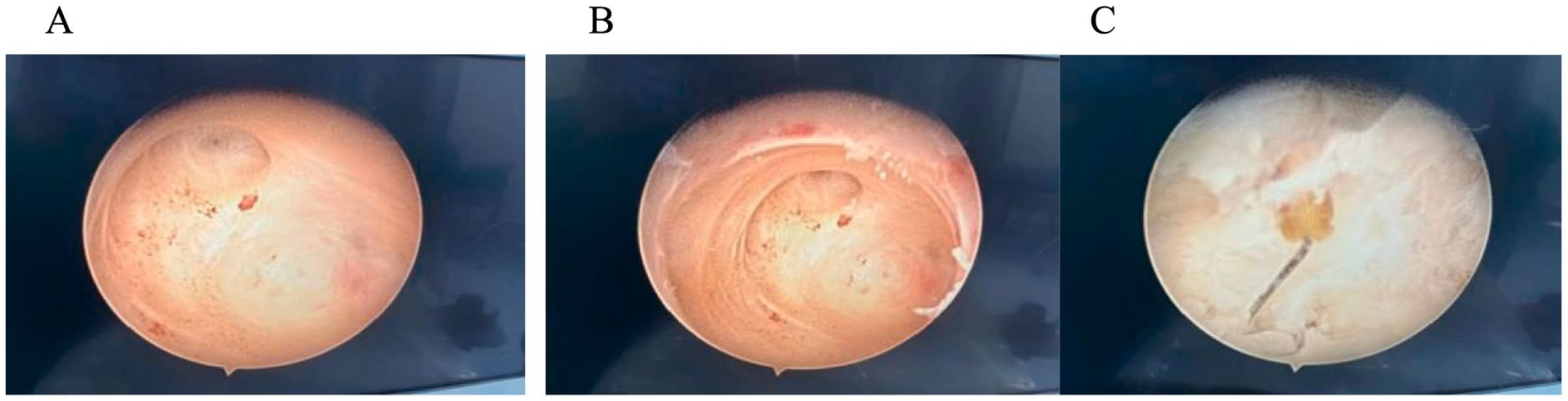
**(A)** Hysteroscopy: no obvious IUD shadow was observed in the cervical canal or uterine cavity. **(B)** In the endometrium, local white scar-like adhesions were observed in the right anterior wall of the fundus. **(C)** Faintly visible metal wire.

**Figure 3:**
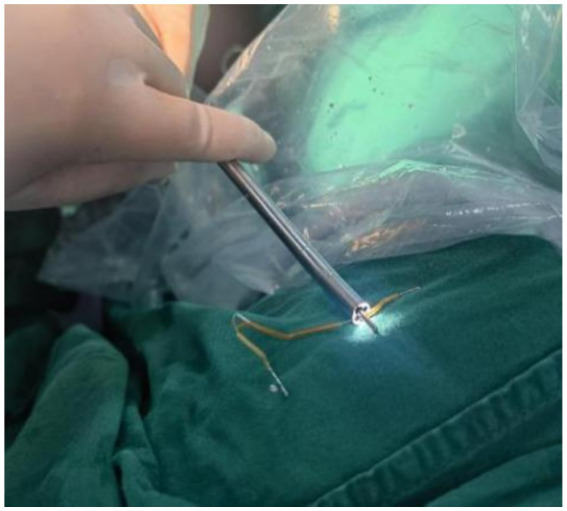
“V” IUD removed during surgery.

**Figure 4:**
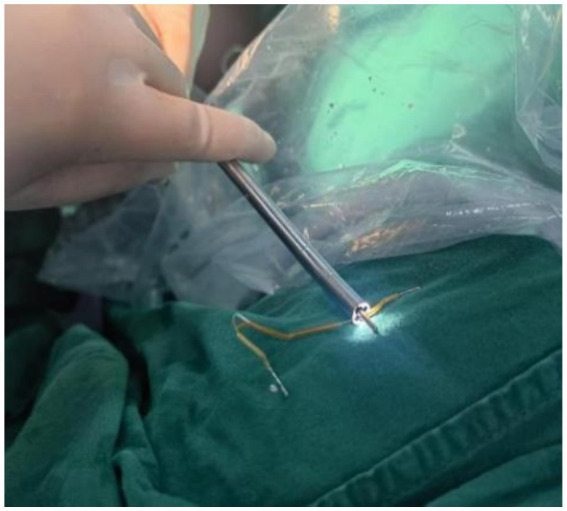
After IUD removal, hysteroscopy was performed again, and no significant abnormalities were observed in utero.

Hysteroscopic exploration was performed first after adequate communication and discussion with the patient, while laparoscopic preparation was also made. Hysteroscopic surgery was conducted under general anesthesia on 1 April 2024. Cervical atrophy and pinpointing of the external orifice were observed during the procedure. A probe was used to explore the uterine cavity under ultrasound monitoring, encountering resistance. After breaking through the resistance, a No. 2 dilator rod was used to explore the middle and posterior positions of the uterus, reaching a depth of 6.5 cm. After dilating to No. 7.5, a small amount of pale brown effusion was released. Hysteroscopy was then performed revealing no obvious IUD shadow in the cervical canal or uterine cavity with maintained pressure at 100 mmHg by inflation instrument (Figure 2A). The endometrium was thin, and local white scar-like adhesions were observed in the right anterior wall of the fundus (Figure 2B). The opening of the right fallopian tube was visible, while the opening of the left fallopian tube was not discernible. Under direct vision, microscissors were employed to cut along the white adhesion band, revealing a faintly visible metal wire (Figure 2C). As the scar-like adhesion band around the IUD was gradually broken from the outside, approximately 1 cm of iron wire and silicone sleeve was exposed. Under direct vision, microforceps and microscissors were used to gradually release the adhesion surrounding the IUD. Microforceps were then used to clamp the IUD. The V-type IUD was slowly pulled and removed (Figure 3). Hysteroscopy again revealed no obvious abnormalities in utero (Figure 4).

**Figure 5:**
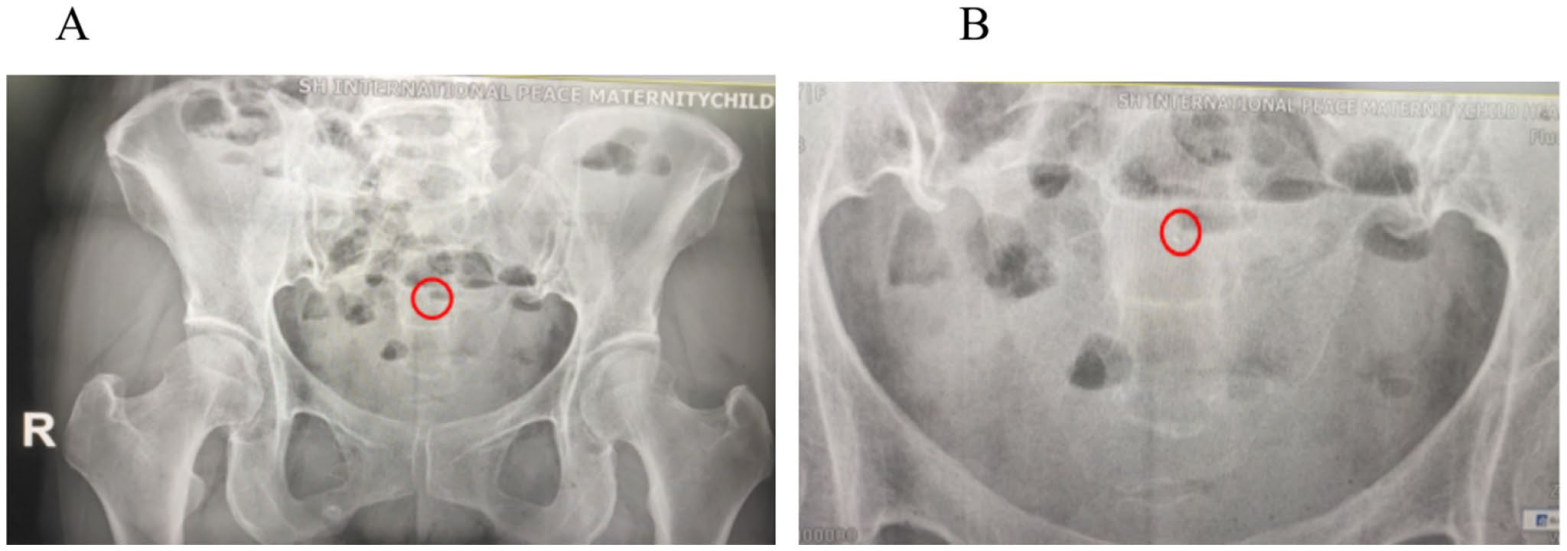
**(A)** Postoperative abdominal plain film: A punctate, slightly high-density shadow approximately 2 mm in length was observed 5.4 cm above the pubic symphysis in the pelvic cavity (red circle). **(B)** An enlarged high-density shadow of 2 mm (red circle).

Intraoperatively and hysteroscopically, no intrauterine residue was observed. Although the removed IUD was intact, it had a patina on the surface. Postoperatively, we reviewed the plain abdominal film to check for any residue. The plain abdominal film revealed a punctate, slightly high-density shadow approximately 2 mm in length located 5.4 cm above the pubic symphysis in the pelvic cavity (Figure 5). After discussion with the patient, the possibility of residual copper rust was considered, noting that there was no obvious abnormality in utero on hysteroscopy at the end of the surgery. Reoperation might not detect high-density shadows, so the patient was scheduled for outpatient follow-up. The patient consented to publication and agreed to follow-up. Currently, more than 5 months after surgery, the patient has reported no abdominal pain, vaginal bleeding, or other discomfort during follow-up.

## Discussion

Technology developments have led to continuous updates in IUD design, an increasing number of users, and an expanding scope of use (7). However, in postmenopausal women who no longer require contraception, the IUD should be removed to reduce complications from organ atrophy. Compared to premenopausal women, removing IUDs in postmenopausal women is more challenging and significantly increased risks. Therefore, this study shares the surgical experience after IUD incarceration to provide new insights for reducing complications, limiting surgical risk, and minimizing the scope of surgery in clinical practice.

## Avoid from patients with IUD who have high risk factors

The incarceration of an IUD is influenced by many factors, which are most closely related to the individual, type of IUD, timing of placement, placement process, and duration of placement.

A history of multiple abortions, abnormal uterine location (e.g., severe uterine flexion and congenital malformations), and fibroids can increase the risk of incarceration (3, 8, 9). In addition, IUD incarceration, especially those in the deep myometrium and extrauterine incarceration, is most commonly associated with “V” shaped IUDs (10). In postmenopausal women, IUD incarceration primarily involves “O” shaped IUDs (11). Finally, the timing of placement also matters; insertion during lactation or following induced abortion heightens the risk (12–14). In addition to the above factors, the longer the placement time, the greater the risk of incarceration (10, 11, 15).

## Discussion on experience in removing incarcerated IUDs

### Comprehensive inspection assessment

A thorough preoperative workup is essential. This includes evaluating the patient’s medical history, IUD type, duration of use, and a careful gynecological examination to assess uterine position and cervical conditions (16). Imaging studies, particularly ultrasound, provide critical insights into the IUD’s intrauterine position and integrity. Pelvic X-rays can clarify the shape and composition of metal-containing IUDs, while CT scans help delineate the IUD’s relationship to the uterine wall and adjacent organs (17, 18). In this case, imaging suggested that part of the IUD reached the uterine serosal surface. A hysteroscopic evaluation was chosen as the initial surgical approach, with laparoscopic surgery as a backup if required (19).

### Adequate preoperative conversation to understand expectations

Comprehensive preoperative counseling is essential to ensure that patients are thoroughly informed about potential risks, including device breakage, residual fragments, and incomplete removal. Transparent and effective communication not only manages patient expectations but also mitigates postoperative psychological distress.

### Adequate preoperative preparation

Adequate cervical preparation, such as with estrogen or prostaglandins, facilitates safer IUD removal, particularly in postmenopausal patients with cervical atrophy or those who have undergone cervical surgeries. In addition, intraoperative ultrasound guidance and the involvement of experienced senior surgeons enhance the likelihood of successful IUD extraction.

### Flexible and gentle surgical procedure

#### Identify the position of the uterus

A comprehensive preoperative ultrasound and gynecological examination are essential to determine the uterine location and exclude the presence of the IUD in the vagina or posterior fornix. Under ultrasound guidance, a probe is used along the uterine axis to measure the depth of the uterine cavity. In patients with a history of cesarean section, abnormal cervical canal, or prior cervical surgery, the length of the cervical canal should be carefully evaluated to avoid excessive force. If perforation occurs during the procedure, the surgery should be immediately halted to assess the perforation’s location, depth, and any potential damage to surrounding organs, with laparoscopic or exploratory laparotomy performed if necessary. In addition, the uterine position may shift after clamping the anterior or posterior cervical lips with forceps. For a uterus with extreme anteversion and flexion, the posterior lip should be clamped, whereas for extreme retroversion and retroflexion, the anterior lip can be clamped. Uterine flexion can be reduced by appropriate traction of the cervical forceps, but the process should be performed gently.

#### Reasonable and skillful use of device

To understand the position of the uterus, after successful cavity exploration, a probe or removal hook and curette can be used after the cervix is dilated to determine the position of the IUD. If the IUD is not detected, hysteroscopy can be used to evaluate the number and position of the IUD (20), the presence or absence of incarceration, and the depth of incarceration. For IUDs primarily located within the uterine cavity, hooks and forceps facilitate gentle extraction. In cases of extensive incarceration or limited mobility, scissors are used to bluntly and sharply dissect surrounding tissues. Considering postmenopausal uterine atrophy, myometrial thickness and distance from the IUD to the uterine serosal surface can be understood using ultrasound and CT before surgery. During surgery, the depth of separation can be measured in relation to the tip length of scissors, forceps, and other instruments used to manipulate the uterine cavity to avoid perforation due to excessive separation distance.

#### Flexible surgical procedures

For patients who have no palpable IUD, no IUD is visible hysteroscopically, and a preoperative examination still suggests that the IUD is in the uterine cavity, and the uterine cavity tissue can be separated appropriately first. In this case, the patient had intrauterine adhesions, and no obvious IUD was found in the uterine cavity or cervix. After decomposing the adhesions, metal wires were faintly visible during surgery. In addition, after the IUD is visualized under hysteroscopy, the decomposition of adhesions should be gradually separated from the outside to the inside, following the direction of the IUD to control the depth and direction of separation. Once the IUD position is loosened, it can be removed by hooking part of the IUD in the uterine cavity and gently pulled out of the cervix with vascular forceps. When one end is tight, it should be cut near the external cervical os, the other end is clamped with straight vascular forceps, and the IUD wire can be pulled slowly. After removal, it is necessary to check for completeness, and when both ends are tight, avoid strong pulling. The patient should be transferred for laparoscopy if no IUD was found under hysteroscopy.

#### Various surgical methods

When IUD incarceration is deep, perforation occurs during removal, and organ injury cannot be excluded or ectopic to a location other than the uterus, and laparoscopic or open surgery should be performed promptly and decisively. There have been case reports of IUDs ectopically located in the rectum (21), so if laparoscopic exploration does not reveal the IUD, anal examination or even enteroscopy should not be overlooked.

#### Evaluate pros and cons and stop in a timely manner

For IUD incarceration, most of the IUD wires pulled out can be cut off, and the two broken ends are left approximately 1 cm at the external cervical os. Surgery can be performed within 7 days of clean menstruation the next month (22). For small amounts of residual material incarcerated in the muscular layer that cannot be removed, postoperative follow-up can be performed. The advantages and disadvantages should be thoroughly assessed during surgery and stopped if necessary (23, 24). The risk of complications such as perforation should not be increased solely to pursue surgical outcomes, and a combined hysteroscopic and laparoscopic approach can be used if necessary (25). A 2018–2022 retrospective cohort study on HELIYON was performed in 2022 (26). The study included 135 patients with ring breaks, 41 with persistent ring breaks, and 82 with spontaneous expulsion, with a mean time to expulsion of 45 days. Therefore, in patients with partial residual ring breaks, the decision to re-operate should be made after a full assessment of the pros and cons.

## Conclusion

In postmenopausal patients, the removal of intrauterine devices is more challenging due to uterine atrophy and the presence of adhesions. Comprehensive preoperative evaluation, patient counseling, and imaging guidance are essential to minimize surgical risks. For incarcerated IUDs, initiating less invasive hysteroscopic techniques while maintaining surgical flexibility is recommended. If necessary, promptly transitioning to laparoscopic or open surgery ensures patient safety. The primary objective is successful removal with minimal trauma, underscored by meticulous planning, precise technique, and adaptability.

## Data Availability

The original contributions presented in the study are included in the article/supplementary material, further inquiries can be directed to the corresponding author.

## References

[1] Zeyi C. Chinese obstetrics and Gynaecology. Beijing: People's Health Publishing House (2008).

[2] Kaislasuo J Suhonen S Gissler M Lahteenmaki P Heikinheimo O. Intrauterine contraception: incidence and factors associated with uterine perforation-a population based study. Hum Reprod. (2012) 27:2658–63. doi: 10.1093/humrep/des246, PMID: 22763376

[3] Heinemann K Reed S Moehner S do Minh T. Risk of uterine perforation with levonorgestrel-releasing and copper intrauterine devices in the European active surveillance study on intrauterine devices. Contraception. (2015) 91:274–9. doi: 10.1016/j.contraception.2015.01.007, PMID: 25601352

[4] Cetinkaya K Kumtepe Y Ingec M. Minimally invasive approach to cases of lost intra-uterine device: a 7-year experience. Eur J Obstet Gynecol Reprod Biol. (2011) 159:119–21. doi: 10.1016/j.ejogrb.2011.07.003, PMID: 21821341

[5] Kho KA Chamsy DJ. Perforated intraperitoneal intrauterine contracep tive devices: diagnosis, management, and clinical outcomes. J Minim Invasive Gynecol. (2014) 21:596–601. doi: 10.1016/j.jmig.2013.12.123, PMID: 24462588 PMC6661232

[6] Huang X Zhong R Zeng L He X Deng Q Peng X. Chronic nodules of sigmoid perforation caused by incarcerated intrauterine contraception device. Medicine. (2019) 98:e14117. doi: 10.1097/MD.0000000000014117, PMID: 30681572 PMC6358395

[7] ESHRE Capri Workshop Group. Intrauterine devices and intrauterine systems. Hum Reprod Update. (2008) 14:197–208. doi: 10.1093/humupd/dmn003 18400840

[8] Yabluchanskiy A Iyer RP Flynn F Cates CA Lindsey ML Han HC. Building a better infarct: modulation of collagen cross-linking to increase infarct stiffness and reduce left ventricular dilation post-myocardial infarction. Mol Cell Cardiol. (2015) 85:229–39. doi: 10.1016/j.yjmcc.2015.06.006, PMID: 26080361 PMC4530076

[9] Caliskan E Oztürk N Dilbaz BO Dilbaz S. Analysis of risk factors associated with uterine perforation by intrauterine devices. Eur J Contracept Reprod Health Care. (2003) 8:150–5. doi: 10.1080/ejc.8.3.150.155, PMID: 14667326

[10] Jiang J Bian S Li S Wang S. Risk factors for intrauterine device embedment in postmenopausal women: an analysis of 731 participants undergoing hysteroscopy. Menopause. (2023) 30:717–22. doi: 10.1097/GME.0000000000002191, PMID: 37162346 PMC10309103

[11] Rowlands S Oloto E Horwell DH. Intrauterine devices and risk of uterine perforation: current perspectives. Open Access J Contracept. (2016) 7:19–32. doi: 10.2147/OAJC.S85546, PMID: 29386934 PMC5683155

[12] Godfrey EM Whiteman MK Curtis KM. Treatment of unscheduled bleeding in women using extended-or continuous-use combined hormonal contraception: a systematic review. Contraception. (2013) 87:567–75. doi: 10.1016/j.contraception.2012.08.005, PMID: 23044386

[13] Anthony MS Reed SD Armstrong MA Getahun D Gatz JL Saltus CW. Design of the Association of uterine perforation and expulsion of intrauterine device study: a multisite retrospective cohort study. Am J Obstet Gynecol. (2021) S0002-9378:00026-0. doi: 10.1016/j.ajog.2021.01.003 33460585

[14] Tabatabaei F Masoumzadeh M. Dislocated intrauterine devices: clinical presentations, diagnosis and management. Eur J Contracept Reprod Health Care. (2021) 26:160–6. doi: 10.1080/13625187.2021.1874337, PMID: 33555216

[15] Agacayak E Tunc SY Icen MS Oguz A Ozler A Turgut A. Evaluation of predisposing factors, diagnostic and treatment methods in patients with translocation of intrauterine devices. J Obstet Gynaecol Res. (2015) 41:735–41. doi: 10.1111/jog.12620, PMID: 25421253

[16] Tabatabaei F Hosseini STN Hakimi P Vejdani R Khademi B. Risk factors of uterine perforation when using contraceptive intrauterine devices. BMC Womens Health. (2024) 24:538. doi: 10.1186/s12905-024-03298-3, PMID: 39334324 PMC11428400

[17] Kaislasuo J Suhonen S Gissler M Lähteenmäki P Heikinheimo O. Uterine perforation caused by intrauterine devices: clinical course and treatment. Hum Reprod. (2013) 28:1546–51. doi: 10.1093/humrep/det074, PMID: 23526304

[18] Kaislasuo J Heikinheimo O Lähteenmäki P Suhonen S. Menstrual characteristics and ultrasonographic uterine cavity measurements predict bleeding and pain in nulligravid women using intrauterine contraception. Hum Reprod. (2015) 30:1580–8. doi: 10.1093/humrep/dev102, PMID: 25990577

[19] Watad H Ifrach U Stockheim D Yulzari V Meron OC Blank M. The contradictive findings between ultrasound, hysteroscopy and cytokines in women with nonhormonal IUDs suffering from menorrhagia: a prospective study. Arch Gynecol Obstet. (2024) 309:2057–62. doi: 10.1007/s00404-024-07457-7, PMID: 38492083 PMC11018669

[20] Varlas VN Meianu AI Rădoi AI Balescu I Bacalbasa N Varlas RG. Intrauterine contraceptive device migrated in the urinary tract: case report and extensive literature review. J Clin Med. (2024) 13:4233. doi: 10.3390/jcm13144233, PMID: 39064273 PMC11278257

[21] Ples L Sima RM Moisei C Ionescu CA. An intrauterine contraceptive device: where did we find it after 29 years of insertion? A case report. J Pak Med Assoc. (2017) 67:131–3. 28065971

[22] Chinese Medical Association Family Planning Section. Technical guidelines for postmenopausal intrauterine device removal. Chinese J Obstetrics Gynaecol. (2019) 54:649–53.

[23] Restaino S Pellecchia G Arcieri M Bogani G Taliento C Greco P. Management for Cervical Cancer Patients: a comparison of the guidelines from the international scientific societies (ESGO-NCCN-ASCO-AIOM-FIGO-BGCS-SEOM-ESMO-JSGO). Cancers. (2024) 16:2541. doi: 10.3390/cancers16142541 39061181 PMC11274772

[24] Restaino S Poli A Arcieri M Martina MD Scambia G Driul L. Discovery of a missing intrauterine system in the peritoneal cavity during cervical cancer surgery: a case report. Acta Biomed. (2024) 95:e2024038

[25] Giampaolino P Della Corte L Di Spiezio SA Zizolfi B Manzi A De Angelis C. Emergent laparoscopic removal of a perforating intrauterine device during pregnancy under regional anesthesia. J Minim Invasive Gynecol. (2019) 26:1013–4. doi: 10.1016/j.jmig.2019.03.012, PMID: 30914327

[26] Cánovas E Beric D Jara R Cazorla E. Intrauterine contraceptive device rupture. Follow-up of a retrospective cohort and clinical protocol. RUDIUS study. Heliyon. (2022) 8:e08751. doi: 10.1016/j.heliyon.2022.e08751, PMID: 35071815 PMC8762393

