# Supplementary figures and images for: Removal of an incarcerated intrauterine device reaching the serosal surface of the uterus by hysteroscopy alone: a case report
Source: Front Med (Lausanne). 2025 Jan 7;11:1486745. doi: 10.3389/fmed.2024.1486745 (PMC11747293; doi:10.3389/fmed.2024.1486745)

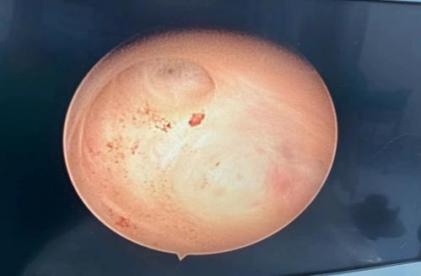

Supplement: Supplementary file 1 [file Image_1.jpeg]

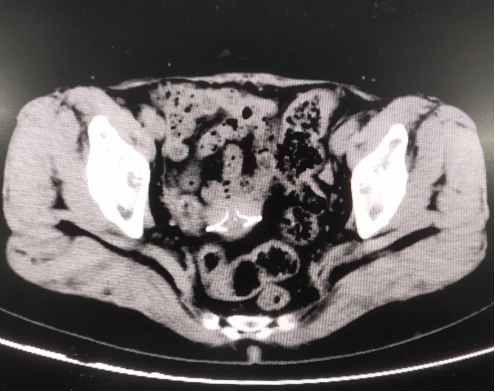

Supplement: Supplementary file 2 [file Image_2.jpeg]

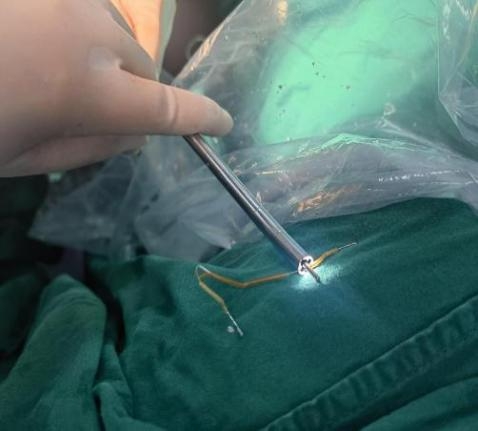

Supplement: Supplementary file 3 [file Image_3.jpeg]
